# First Annual Report of the Visitors of the Cincinnati Eye Infirmary

**Published:** 1829-02

**Authors:** 


### II. Original.

### FIRST ANNUAL REPORT OF THE VISITORS OF THE CINCINNATI EYE INFIRMARY.

The Visitors of the Cincinnati Eye Infirmary, in obedience to the act establishing the Institution, have the honour to submit to the General Assembly the following *Report:*

The Infirmary projected by Dr. Daniel Drake, in concert with a number of citizens of Cincinnati, began its operations on the first of July, 1827, and was incorporated by the Legislature at the succeeding session.

During the eighteen months that have elapsed, there has been no period when it has not had indigent citizens of Ohio, as patients; some of whom have been cured, others relieved, and others discharged as incurable, from the state in which the intrinsic violence, or the wrong and inefficient treatment of the early stages of their complaints, had left them.

These patients have all been the beneficiaries of the Infirmary. Some of them have been boarded and lodged in places selected by the surgeon; others have received partial assistance of the same kind; and others, only the requisite medicines, according to the degree of their indigence; but all have experienced the gratuitous personal services of the surgeon, according to the original plan and design of the institution.

The funds for these laudable purposes, have been exclusively derived from the voluntary contributions of certain citizens of Cincinnati and its vicinity, who have thus charitably lent their aid, to many unfortunate and needy persons; not only of the city, but also from various parts of the state. To make permanent and adequate provision, for this numerous class of patients, is obviously beyond the means of one portion of the community, and the Visitors, therefore, would respectfully represent to your honourable body, that in their opinion, few subjects of deeper interest, to the people of the state at large, *could* engage the attention of their Representatives, or make a more powerful appeal to the public charity, than the infant Infirmary committed to their charge.

The number of poor persons who labour under diseases of the eye, (in all respects one of the most important organs of body) is far greater than is generally supposed, and more have already required assistance at the Infirmary, than with the slender charity fund, placed at the disposal of the Visitors, could be received.

Many, indeed most of the maladies of the eye, as the Visitors have been advised, are tedious in their cure, and require a long attendance at the Infirmary. An ample fund, therefore, is necessary to the fulfillment of the benevolent design of the founders of the institution, and of the General Assembly, by whom it was incorporated; and the Visitors venture to express the hope, that your honourable body will not be slow in devising such sources of revenue, for the institution, as will enable it to dispense the extensive benefits which, with adequate means, it is so well calculated to confer.

But it is not in reference to paupers only, that the state might be called upon to endow an Infirmary for diseases of the eye. All classes of society are interested in its success; for, as the Visiters are informed and believe, the maladies of the eye and the operations which many of them require, are frequently of such a peculiar and complicated character, as to call for much special preparation, and a greater variety of means, than are possessed by the physicians of the state generally. All persons having such complaints are interested in the full establishment, within the state, of an Infirmary to which they may resort, instead of visiting the distant hospitals and occulists east of the mountains.

The Visitors consider it their duty to state, that for more than three months past, the benefits of the Infirmary have been but partially dispensed, in consequence of a severe burn which the surgeon received on both hands; and which at one time threatened to incapacitate him for many of the duties of his office. While in this situation, the Visitors at his suggestion, were induced to appoint Jedediah Cobb, M. D. a consulting surgeon, and have received his acceptance of the same. They are gratified, however, to be able to say, that no permanent decripitude of a serious kind, will follow the casualty to which they have referred; but still the union of another professional gentleman with the Infirmary, cannot fail to increase its claims on the confidence of the community.

All which is respectfully submitted.

J. L. Wilson, President,
Davis B. Lawler, Secretary,
Wm. M. Walker, Treasurer,
Peyton S. Symmes,
J. P. Foote,
Wm. Burke,
Lewis Howel,
John Locke.

*Cincinnati, Ohio, December 1st,* 1828.

